# Construction of a dense genetic linkage map and mapping quantitative trait loci for economic traits of a doubled haploid population of *Pyropia haitanensis* (Bangiales, Rhodophyta)

**DOI:** 10.1186/s12870-015-0604-4

**Published:** 2015-09-21

**Authors:** Yan Xu, Long Huang, Dehua Ji, Changsheng Chen, Hongkun Zheng, Chaotian Xie

**Affiliations:** College of Fisheries, Jimei University, Xiamen, 361021 PR China; Biomarker Technologies Corporation, Beijing, 101300 PR China

## Abstract

**Background:**

*Pyropia haitanensis* is one of the most economically important mariculture crops in China. A high-density genetic map has not been published yet and quantitative trait locus (QTL) mapping has not been undertaken for *P. haitanensis* because of a lack of sufficient molecular markers. Specific length amplified fragment sequencing (SLAF-seq) was developed recently for large-scale, high resolution de novo marker discovery and genotyping. In this study, SLAF-seq was used to obtain mass length polymorphic markers to construct a high-density genetic map for *P. haitanensis*.

**Results:**

In total, 120.33 Gb of data containing 75.21 M pair-end reads was obtained after sequencing. The average coverage for each SLAF marker was 75.50-fold in the male parent, 74.02-fold in the female parent, and 6.14-fold average in each double haploid individual. In total, 188,982 SLAFs were detected, of which 6731 were length polymorphic SLAFs that could be used to construct a genetic map. The final map included 4550 length polymorphic markers that were combined into 740 bins on five linkage groups, with a length of 874.33 cM and an average distance of 1.18 cM between adjacent bins. This map was used for QTL mapping to identify chromosomal regions associated with six economically important traits: frond length, width, thickness, fresh weight, growth rates of frond length and growth rates of fresh weight. Fifteen QTLs were identified for these traits. The value of phenotypic variance explained by an individual QTL ranged from 9.59 to 16.61 %, and the confidence interval of each QTL ranged from 0.97 cM to 16.51 cM.

**Conclusions:**

The first high-density genetic linkage map for *P. haitanensis* was constructed, and fifteen QTLs associated with six economically important traits were identified. The results of this study not only provide a platform for gene and QTL fine mapping, map-based gene isolation, and molecular breeding for *P. haitanensis*, but will also serve as a reference for positioning sequence scaffolds on a physical map and will assist in the process of assembling the *P. haitanensis* genome sequence. This will have a positive impact on breeding programs that aim to increase the production and quality of *P. haitanensis* in the future.

**Electronic supplementary material:**

The online version of this article (doi:10.1186/s12870-015-0604-4) contains supplementary material, which is available to authorized users.

## Background

*Pyropia/Porphyra* is one of the most important marine macroalgae in terms of both its global distribution and economic importance. According to Yoshida et al. [1] and Sutherland et al. [2], over 130 species of *Pyropia/Porphyra* have been described worldwide. Farming and processing of *Pyropia* have generated the largest seaweed industries in East Asian countries, such as China, Japan, and South Korea [3, 4]. In China, two major cultivars, *Pyropia yezoensis* Ueda and *Pyropia haitanensis* Chang et Zheng, are distributed in North China and South China, respectively. *P. haitanensis*, as a typical warm, temperate zone species originally found in the south of China, has been extensively cultured in Fujian, Zhejiang and Guangdong Provinces of China for more than 50 years. Its output accounts for about 75 % of the total production of cultivated *Pyropia* in China [4, 5].

Through years of genetic study and breeding, some improved varieties of *P. haitanensis* have been obtained and cultivated widely [6–8]. To some degree, this enhanced the cultivation of *Pyropia* and promoted the industrial development of this economic seaweed. However, *P. haitanensis* cultivation still faces many problems. First, to date, the cultivation of *P. haitanensis* in some areas still relies on natural populations, with very limited germplasm development and genetic improvement. Second, the genetic basis for most of the traits related to commercial production is still undetermined, and we lack varieties of *P. haitanensis* with high yield or high quality [9]. Thus, it is highly desirable to carry out breeding studies and to cultivate elite species to raise the industrial economic efficiency and expand the scale of *P. haitanensis* cultivation.

Plant breeding is a dynamic area of applied science. It relies on genetic variation and uses selection to improve plant characteristics that are of interest to the grower and consumers; however, this is a time-consuming and labor-intensive field evaluation process. The development of high yield or high quality varieties is a major goal in *Pyropia* breeding; however, traits related to production or quality of *P. haitanensis*, such as frond length (FL), frond width (FW), frond thickness (FT), fresh weight (W), and growth rates, are quantitative characteristics [9–11]. It is believed that these complex traits are controlled by multiple genes and are susceptible to environmental changes [9, 12]. Methods to analyze such complex traits, particularly to uncover their potential genetic bases, are of prime importance for breeding purposes. In recent years, with the availability of molecular markers to develop well-saturated genetic maps and statistical methodology to dissect complex traits, mapping of quantitative trait loci (QTLs) has proved to be an effective approach to study the genetic architecture of quantitative traits. QTL analysis is a powerful strategy to identify underlying genes and elements when combined with map-based cloning, because it allows the estimation of the QTL number, their genomic position, and their genetic effects [13]. This method has been applied successfully to most farm animal species, crops and some aquaculture species [13–15]. However, among economically important seaweeds, the method has only been used to analyze the genetic bases of two quantitative traits (FL and FW) of *Laminaria japonica* and located their genetic loci on a high-density map [16].

The efficiency of QTL mapping largely depends on the marker density of the genetic map. For a given trait in a particular population, increasing the marker density can increase the resolution of the genetic map, thus enhancing the precision of QTL mapping [17]. Traditionally, the development of markers such as simple sequence repeats (SSRs), restriction fragment length polymorphisms (RFLPs) and amplified fragment length polymorphisms (AFLPs) was a costly, low-throughput and iterative process that involved time-consuming cloning and primer design steps that could not easily be parallelized. Scoring of marker panels across target populations was also expensive and laborious. The development of next generation sequencing technology has make it possible to discover huge numbers of markers rapidly throughout the genome to construct high-density genetic maps and make genotyping easier. Recently, several cost effective methods of markers discovery and high-throughput genotyping were developed, such as RAD-seq (restriction site-associated sequencing), double digest RAD-seq, GBS (two-enzyme genotyping-by-sequencing), and SLAF-seq (specific length amplified fragment sequencing) [17, 18]. Among them, SLAF is measured by sequencing the paired-ends of sequence-specific restriction fragment lengths. SLAF involves fragment length selection but not random interruption; therefore, its repeatability and accuracy are better than RAD and GBS [18, 19]. SLAF has been used successfully to create genetic maps for common carp [18], sesame [20], kiwifruit [21] and soybean [19].

In previous works, the first genetic linkage map of *P. haitanensis* was constructed [22], and some quantitative traits were analyzed [9]; however, a high-density genetic map has not been published yet and QTL mapping has still not been undertaken for *P. haitanensis* because of a lack of sufficient molecular markers. Therefore, in this study, we constructed a higher density genetic map for *P. haitanensis* based on the recently developed SLAF-seq approach and then mapped QTLs controlling certain economic traits of *P. haitanensis*.

## Results

### Genotyping of a double haploid (DH) population based on SLAF-seq

The DH population was genotyped using SLAF-seq technology. According to the results of a pilot experiment, Hae III and Hpy166II were chosen to construct the SLAF library. The library comprised SLAF fragments that were 264–464 bp in size. After high-throughput sequencing, 120.33 Gb of data containing 75.21 M pair-end reads was obtained, with each read being 80 bp in length. The Q30 (representing a quality score of 30, indicating a 0.1 % chance of an error, and thus 99.9 % confidence) ratio was 78.52 % and guanine-cytosine (GC) content was 53.19 %. Among these high quality data, approximately 1.6 Gb were from the male parent (10,021,701 reads) and approximately 1.4 Gb were from the female parent (9,291,420 reads); the average read numbers of the 100 individuals in the DH population was 542,720.

**Fig. 1:**
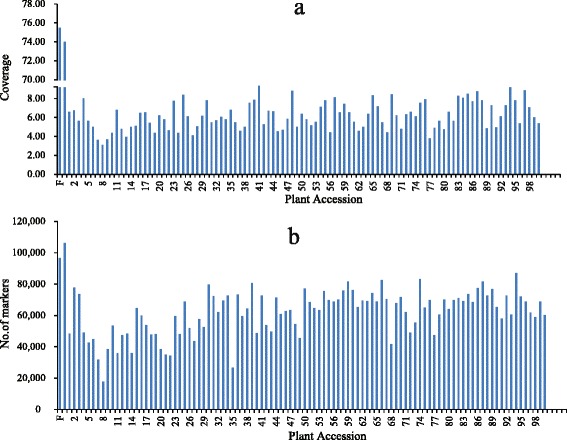
Coverage and number of markers for each double haploid (DH) individual and their parents. The x-axes in (**a** and **b**) indicate the plant accession, including the female parent and the male parent, followed by each of the DH individuals; the y-axes indicate coverage in (**a**) and number of markers in (**b**)

The numbers of SLAFs in the male and female parents were 96,652 and 106,272, respectively. The read numbers for the SLAFs were 7,296,857 and 7,865,906 in the male and female parents, respectively. The average coverage for each marker was 75.50-fold in the male parent and 74.02-fold in the female parent. In the DH population, the numbers of SLAF markers in each individual ranged from 17,751 to 87,038 (average of 61,136). The read numbers for SLAFs ranged from 54,860 to 802,063 (average of 384,760), and the coverage ranged from 3.09-fold to 9.35-fold (average of 6.14-fold) (Fig. 1).

Among the 188,982 detected high-quality SLAFs, 8553 were polymorphic, giving a polymorphism rate of only 4.53 % (Table 1). Of the 8553 polymorphic SLAFs, 2372 were classified into eight segregation patterns (Table 2). The genotype of the DH line is aa or bb; therefore, only the aa × bb segregation pattern in the DH population was used to construct the genetic map, and 1748 markers fell into this class. Among these 1748 markers, after filtering out the markers with average sequence depths less than 10-fold in the parents, an integrity < 30 % and those showing segregation distortion, only two markers could be used for genetic map construction. Thus, these polymorphic SLAF markers were not suitable for genetic map construction.

**Table 1:** SLAF markers mining results

Type	Polymorphic SLAF	Non-Polymorphic SLAF	Repetitive SLAF	Total SLAF
Number	8553	180,394	35	188,982
Percentage	4.53 %	95.46 %	0.02 %	100 %

**Table 2:** Number of polymorphic SLAF markers for the eight segregation patterns

Type	Polymorphic SLAF
No_P_M	6181
ab × cd	8
ef × eg	6
hk × hk	273
lm × ll	219
nn × np	124
aa × bb	1748
aa × cc	19
cc × ab	10

**Fig. 2:**
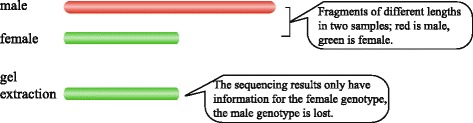
Schematic diagram of the production of length polymorphic (LP) markers

Exploiting the variation in restriction sites, enzyme digestion can produce fragments of different lengths in the two parents, and using SLAF-seq through gel extraction screening for fragments of a certain length, the same locus in the sequencing data will detect only one genotype, as shown in Fig. 2. As a result, during data analysis in the project, a large number of fragments of different lengths and one genotype only are detected, and these length polymorphic (LP) fragments were regarded as non-polymorphic SLAFs in the conventional analysis. Thus, the 180,394 non-polymorphic SLAFs in this project could be used as LP markers to construct a genetic map.

For the 180,394 non-polymorphic SLAFs, the SLAFs that were present only in the female parent or the male parent were first screened. The map population is DH; therefore, the SLAFs which were only present in the female parent were typed as aa, and the genotypes of offspring in which the SLAFs could be detected were also typed as aa. The genotypes of the male parent and offspring in which the SLAFs could not be detected were typed as bb. The SLAFs only present in the male parent were typed as bb, and the genotypes of offspring in which the SLAFs could be detected were also typed as bb; however, the genotypes of male parent and offspring in which the SLAFs could not be detected were typed as aa. Consequently, 86,190 LP markers were obtained. To ensure the quality of the genetic map, the 86,190 LP markers were further screened based on three criteria: i) the sequencing depth in the female parent or in the male parent must be larger than 10×; ii) the average sequencing depth in the offspring must larger than 3×; and iii) no significant segregation distortion must be present (*P* < 0.05). Ultimately, 6731 LP markers satisfied the criteria and were used to construct the genetic map.

### Basic characteristics of the genetic map

**Fig. 3:**
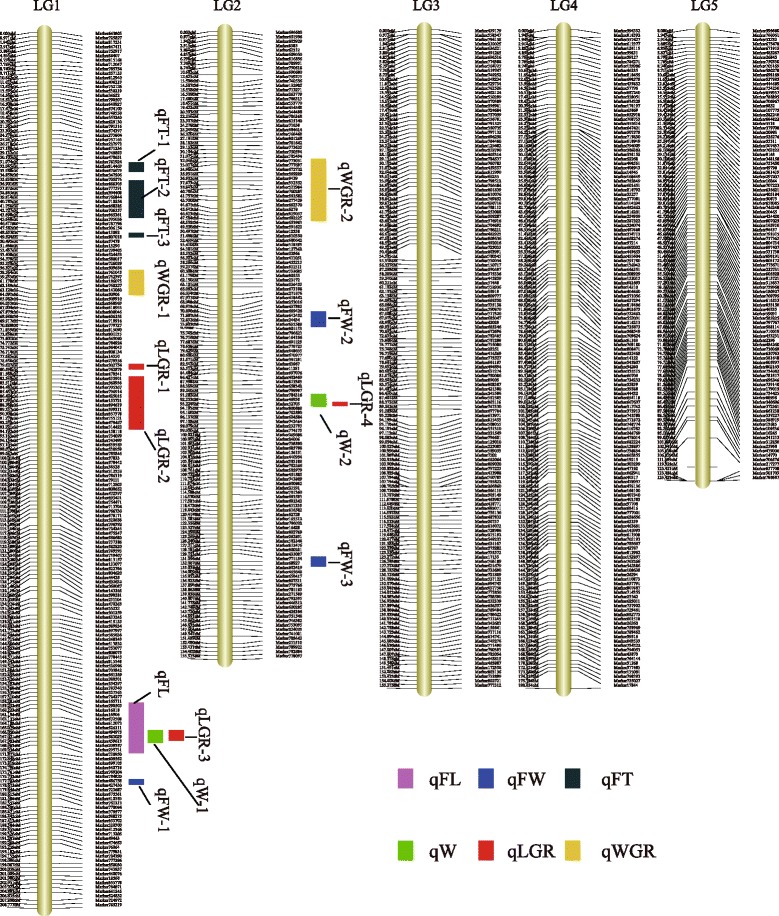
High-density linkage map for *P. haitanensis* and QTL locations in the map for six economically important traits. FL, frond length; FW, frond width; FT, frond thickness; W, fresh weight; LGR, frond length growth rate; WGR, fresh weight growth rate

After linkage analysis, 4550 (Additional file 1: Table S1) of the 6731 (Additional file 2: Table S2) LP markers were mapped onto the genetic map, while the other 2181 markers failed to be linked to any group. The 4550 markers were distributed on the five linkage groups (Additional file 3: Table S3). For the obtained linkage groups that contained many redundant markers that provided no new information, the bin-markers approach was used to combine them into bins that showed a unique segregation pattern and were separated from adjacent bins by a single recombination event into one bin (Additional file 4: Table S4). Through this step, the final genetic map included 740 bins and was 874.33 cM in length, with an average distance of 1.18 cM between adjacent bins (Fig. 3, Table 3). As shown in Table 3, the largest linkage group (LG) was LG1 with 198 bins, a length of 208.78 cM, and an average distance of only 1.05 cM between adjacent bins. The smallest LG was LG5, with 102 bins, a length of 140.02 cM, and an average distance of 1.37 cM between adjacent bins. The degree of linkage between bins was reflected by “Gap < 2”, which ranged between 94.06 % and 100 %, with an average value of 97.87 %. The largest gap on this map was 7.83 cM in LG5.

**Table 3:** Summary of the five genetic linkages groups for *P. haitanensis*

Linkage group ID	Total marker	Total bin	Total distance (cM)	Average distance (cM)	Max gap (cM)	Gaps < 2
LG1	1,154	198	208.78	1.05	1.94	100 %
LG2	1,013	142	155.37	1.09	1.94	100 %
LG3	1,025	149	167.19	1.12	3.89	99.32
LG4	683	149	202.98	1.36	4.87	95.95
LG5	675	102	140.02	1.37	7.83	94.06
Total	4,550	740	874.33	1.18	7.83	97.87

“Gap < 2” indicates the percentages of gaps in which the distance between adjacent bin markers was less than 2 cM

### Visualization and evaluation of the genetic map

Haplotype maps and a heat map were used to evaluate the quality of the genetic map. A haplotype map reflects the proportion of double crossovers, which suggested genotyping errors. Haplotype maps (Additional file 5) were generated for each of the 100 lines of the DH population and for the parental controls, using the 4550 LP markers, as described by West et al. [23]. The haplotype maps intuitively displayed the recombination events of each line (Additional file 5). Most of the recombination blocks were clearly defined. Less than 0.1 % had heterozygous fragments, and less than 0.6 % were missing. Although high frequency recombination events did occur in the DHs, all linkage groups were distributed uniformly, only a few sites showing heterozygosity were present. Therefore, the DH population was well purified and suitable for genetic analysis.

The heat map reflects the relationship of recombination between markers from one linkage group, which can be used to find ordering errors. Heat maps were created to evaluate the genetic map quality using pair-wise recombination values for the 4550 LP markers (Additional file 6). Visualization of the heat map showed that, in general, the LGs performed well.

### Phenotypic traits

Six economically important traits, FL, frond length; FW, frond width; FT, frond thickness; W, fresh weight; LGR, frond length growth rate; WGR, fresh weight growth rate, of the parents and the 100 DH lines were measured (Additional file 7: Table S5). The phenotypic values of the traits measured in the DH population were continuously distributed. The coefficient of variation for the six traits was between 20.43 % and 50.35 % (Table 4). The asymptotic significance of a one-sample Kolmogorov-Smirnov test showed that the frequency of the six traits in the DH population was in accordance with a normal distribution (*P*_ks_ > 0.05) (Table 4), indicating that all the measured traits were quantitatively inherited.

**Table 4:** Performance of characters in the DH population and its parents

Character	Male parent	Female parent	DH population	Coefficient of variation	Asymptotic significance of one-sample Kolmogorov-Smirnov test (*P*_ks_)
FL (cm)	23.19 ± 3.70**	34.08 ± 3.90**	25.37 ± 11.55	45.57 %	0.159
FW (mm)	6.82 ± 1.12**	2.20 ± 0.25**	5.86 ± 2.00	34.03 %	0.277
W (mg)	78.43 ± 16.66**	39.99 ± 7.22**	75.40 ± 43.24	57.35 %	0.398
FT (μm)	33.38 ± 1.96**	26.25 ± 1.77**	32.78 ± 6.89	20.43 %	0.758
LGR (%)	17.06 ± 1.06**	20.17 ± 0.94**	16.64 ± 4.84	29.07 %	0.944
WGR (%)	34.86 ± 2.74**	24.20 ± 2.00**	30.31 ± 7.02	23.16 %	0.057

Data are the mean ± SD (n = 30); *t* tests were used to analyze differences between parents; **highly significant (*P* ≤ 0.01)

### QTL analysis

Based on the high-density genetic map, QTLs underlying the six economically important traits, FL, FW, FT, W, LGR and WGR were identified. The threshold of the logarithm of odds (LOD) scores to evaluate the statistical significance of the QTL effects was determined using 1000 permutations. As a result, intervals with a LOD value above 2.5 were detected as effective QTLs, using the winQTLCart software. According to the threshold, 15 QTLs associated with the six traits investigated were identified on LG1 and LG2; no QTLs were found on the other LGs (Fig. 3, Table 5). Among the 15 QTLs, one was associated with FT, three each were associated with FW and FT, two each were associated with W and WGR, and four were associated with LGR. The minimum and maximum LOD scores were recorded as 2.64 and 4.54, respectively. The value of phenotypic variance explained (PVE) by each individual QTL ranged from 9.59 to 16.61 %. The minimum and maximum confidence intervals of the QTLs were 0.97 cM and 16.51 cM, respectively (Table 5).

**Table 5:** Detail of QTLs related to economic traits

Trait	QTL	LOD^a^	Linkage group ID	IC (cM)^b^	Marker number^c^	PVE^d^	ADD^e^
FL	qFL	4.54	1	158.29–170.94	11	16.61 %	126.87
FW	qFW-1	2.64	1	177.71–178.68	1	9.67 %	23.84
	qFW-2	3.89	2	69.93–73.81	4	12.2 %	88.40
	qFW-3	4.21	2	130.16–133.05	3	16.7 %	74.85
FT	qFT-1	2.92	1	31.08–34	2	12.11 %	51.38
	qFT-2	4.04	1	35.94–45.65	9	13.84 %	163.09
	qFT-3	2.67	1	48.56–49.53	1	11.59 %	24.53
W	qW-1	3.39	1	166.06–168.97	3	11.61 %	54.23
	qW-2	2.96	2	90.35–92.26	2	14.52 %	38.35
LGR	qLGR-1	2.93	1	79.64–80.61	1	10.79 %	25.28
	qLGR-2	3.75	1	82.55–94.2	12	12.51 %	193.06
	qLGR-3	3.61	1	166.06–168	2	12.14 %	43.44
	qLGR-4	2.65	2	91.29–92.26	1	9.59 %	23.82
WGR	qWGR-1	3.51	1	56.33–63.13	5	12.8 %	117.14
	qWGR-2	4.38	2	31.08–47.59	14	13.35 %	275.31

^a^LOD indicates the logarithm of odds score

^b^IC indicates the interval of confidence in centimorgans

^c^Marker number indicates the number of bin markers in the confidence interval

^d^PVE indicates the phenotypic variance explained by individual QTL

^e^ADD indicates the additive effect value

## Discussion

### Features of the high-density map of *P. haitanensis*

Linkage maps, especially high-density ones, play an important role in the study of genetics and genomics. In this study, we employed the recently developed SLAF-seq approach to achieve the first, rapid mass discovery of LP markers for *P. haitanensis*. Using these newly developed LP markers, a high-density genetic map of *P. haitanensis* was constructed and its characteristics were investigated for a DH population. In this map, the LG number was equal to the haploid chromosome number of *P. haitanensis* [24]; however, in the absence of cytological markers, we cannot judge if each linkage group corresponded to each chromosome. The map spans 874.33 cM, with an average number of 148 bins per LG and an average distance of 1.18 cM between adjacent bins (Table 3). The total map length is similar to that of a previously reported *P. haitanensis* genetic map, which spanned 830.6 cM; however, the average distance in the present map is much less than the 10.13 cM previously reported [22]. The markers were distributed evenly on the map, with 97.87 % of the gaps being less than 2 cM and the largest gap being 7.83 cM (Table 3). Visual evaluation of the genetic map was performed using haplotype maps and heat maps, which demonstrated that all linkage groups were distributed uniformly, with only a few sites showing heterozygosity. Thus, we believe that it is a high quality genetic map. Compared with PCR-based methods in the same DH population [22], the sequencing-based high-throughput method produced a more than 8-fold denser genetic map and took only 3 weeks to genotype 100 DHs. Thus, this powerful technique is considerably more efficient, cost-effective and less laborious.

To the best of our knowledge, the genetic map presented in this paper is the first high-density genetic linkage map for *P. haitanensis*, though it is still not saturated. Compared with published genetic linkage maps in other macroalgae, such as *L. japonica* ([25]: average density of 8.0 cM; [26]: average density of 9.4 cM; [27]: average density of 7.91 cM), this *P. haitanensis* map is the densest. The results of this study not only provide mass markers for *P. haitanensis*, but also provide useful data for gene and QTL fine mapping, map-based gene isolation and molecular breeding. The whole genome sequencing of *P. haitanensis* is underway (personal communication), and because our high-density map was constructed based on molecular markers developed at the whole genome level, they will also serve as a reference for positioning sequence scaffolds on the physical map to assist in the assembly process of the *P. haitanensis* genome sequence.

High-density genetic maps of populations with high linkage disequilibrium contain many redundant markers that provide no new information, but do increase the computational requirements of mapping [28]. To address this issue, a bin marker approach was applied to the construction of the high-density genetic map of *P. haitanensis*, one “bin” means a group of markers with a unique segregation pattern that is separated from adjacent bins by one recombination event. The bin-map strategy was efficient for generating ultra-high-density genetic maps and identifying QTLs at high resolution in several crops [28–31]. Compared with conventional molecular markers, such as RFLPs, SSRs or single nucleotide polymorphism markers, bin markers are the most informative and parsimonious set for a given population [28]. In this study, 4550 LP markers were grouped into 740 bins. Although the LP markers in one bin appeared at the same position on this genetic map, their actual physical positions were not at the same location. These markers could be used for different populations, in which they may show different diversities.

### QTLs of economically important traits of *P. haitanensis*

QTLs are chromosomal regions determining a quantitative character that can identify genes affecting economic traits [32]. Hence, through QTL studies, the numbers and effects of genes that determine one quantitative trait can be determined and could be used in selective breeding to accelerate the genetic improvement of this trait. In recent decades, there has been a remarkable increase in the use of QTL mapping as a tool to uncover the genetic control of economic traits in aquaculture species, and such studies have been carried out in more than 20 aquaculture species [15]. The economically important traits of *P. haitanensis*, FL, FW, FT, W, LGR and WGR, are under selection during a breeding program and are controlled by QTLs [9]. This study presents the first example of QTL detection for economic traits in a DH population of *P. haitanensis* using a high-density linkage map and phenotypic data, although these phenotypic data were obtained under only one environment. Fifteen QTLs associated with FL, FW, FT, W, LGR and WGR were identified (Table 5). These results will enable further fine mapping of these QTLs in *P. haitanensis*, eventually identifying the individual genes responsible for these economic traits. The information from these molecular makers could be used in selective breeding programs to increase the production and quality of *P. haitanensis* in the future.

Compared with the low-density map constructed previously, the present high-density genetic map proved to be more powerful for identifying precise QTLs controlling important agronomic traits. Previously, using the low-density map, only seven QTLs were identified and only three showed a PVE as large as 10 % [33]. By contrast, in this study, 15 QTLs were identified and only two showed a PVE of less than 10 % (Table 5). In a previous study, Collard et al. reported that a major QTL is defined as one contributing 10 % or more phenotypic variation [34]. Therefore, thirteen of the QTLs presented in this study may be regarded as major QTLs in *P. haitanensis* breeding programs.

Previous studies in fine mapping and map-based cloning have found that QTLs and genes can exhibit pleiotropic effects on multiple traits, and phenotypically correlated traits are often mapped together [30]. In this study, the co-localizations of QTLs for several traits investigated were clearly observed in some chromosomal intervals; for example, the interval of confidence (IC) of qFL includes the IC of qW-1 and qLGR-3, and the IC of qW-2 includes the IC of qLGR-4. These observations were not surprising, because in the correlation analysis of the quantitative traits of *P. haitanensis*, the traits of FL, W and LGR showed significant positive correlations [9]. One important goal of genomic and genetic studies of plants is to identify important loci and genes that could be used to improve agronomic traits and, thereby, agricultural productivity [12]. Our results provide useful information on target chromosomal intervals for candidate gene analysis and marker-assisted selection breeding, because these intervals could be regarded as hotspots with agronomical importance, although additional studies are needed to confirm these findings. Taking such hotspots based on QTL results as prior chromosomal regions, a strategy has been suggested for candidate gene isolation [35]. The relationship between the genetic bin map and the physical position of LP markers is consistent; therefore, it is easy to anchor the physical interval and find the putative genes in this region. Moreover, the transfer of large chromosomal intervals from a donor parent into a recurrent parent has been proposed [30].

## Conclusions

In this study, the SLAF-seq approach was used for large-scale marker discovery and genotyping to develop a high-density genetic linkage map of *P. haitanensis* from a DH population of 100 lines. Our results suggested that this high-density genetic map is accurate and of high quality. The map was used for QTL mapping to identify chromosomal regions associated with six economically important traits: FL, FW, FT, W, LGR and WGR. Fifteen QTLs (including 13 major QTLs) were identified (one for FT, three for FW and FT, two for W and WGR, and four for LGR). The present study increases our knowledge of the genetic control of these economically important traits of *P. haitanensis*. These data, together with the molecular resources generated herein (e.g., the high-density map and the mass of LP markers), will have a positive impact on future breeding programs that aim to increase the production and quality of *P. haitanensis*.

## Methods

### Construction of map population

A DH population of 100 lines was used to construct the genetic linkage map of *P. haitanensis*. The parental lines used in the hybridization experiment were a wild-type line (♂), YSIII, and a red-type artificial pigmentation mutant line (♀), RTPM. The free-living conchocelis of the wild-type line were established in 1999 from a gametophytic blade collected on the coast of Dongshan Island, Fujian Province, China, and has been maintained in the laboratory. The stock culture was maintained at 21 ± 1 °C under 50-60 μmol · photons m^-2^ s^-1^ (12Light (L):12Dark (D)) provided by cool white fluorescent lamps, by renewing the culture medium (MES) [36] once every month. Free-living conchocelis of the red type artificial pigmentation mutant line of *P. haitanensis* were obtained by treatment of the gametophytic blades of another wild-type with ^60^Co-γ rays [6].

To prepare the DH population, the mature free-living conchocelis of each parent were induced to release conchospores. The conchospores were collected in a 300-mL flask containing 200-mL culture medium and cultured with aeration in an incubator at 25 ± 1 °C under 80 μmol · photons m^-2^ s^-1^ (10 L: 14D) to develop into gametophytic blades, with culture medium renewed every 3 days. After approximately 2 months in culture, healthy gametophytic blades were selected as parents for crossing experiments, and a male and a female blade were co-cultured in a flask until carposporangia appeared. About 2 weeks later, the fertilized female blade was transferred into a new flask and cultured under the same conditions until carpospores were released. The carpospores were collected and grown individually to conchocelis colonies in a test tube. When the conchocelis colonies grew to a certain size, they were fragmented by a homogenizer and continued in culture until the conchospores were released. Culture conditions and methods were the same as described above. Once conchospores were released from the heterozygous conchocelis filaments, they were collected and passed gently through a 50-μm nylon mesh filter, and cultured in Petri dishes containing the culture medium at 25 ± 1 °C under 40 μmol · photons m^-2^ s^-1^ (10 L:14D) to obtain F_1_ gametophytic blades. After 40 days in culture, the F_1_ gametophytic blades were picked out and transferred onto a slide glass to examine the types of F_1_ blades under a light microscope (Nikon SMZ800). Each partial color phenotype F_1_ blade was obtained by a puncher and digested into a single vegetative cell by 2 % snail enzymes dissolved in 2-mol/L glucose liquor. The vegetative cells were then induced to develop into conchocelis (with double the normal amount of chromosomes) by single somatic cell clone cultivation [37], producing the DH population. During processing, 166 color-sectors were gained from 50 F_1_ blades, and only 100 color-sectors were developed into conchocelis.

### DNA extraction

DNA was isolated from free-living conchocelis of each parental line and 100 DH lines. The collected free-living conchocelis were ground into a powder using a high-speed homogenizer, and the DNA was extracted and purified by the Cetyltrimethyl Ammonium Bromide (CTAB) method [38]. The DNA concentration and quality were determined using a DU-600 spectrophotometer (Beckman Coulter, Fullerton, CA, USA) and by electrophoresis through 0.8 % agarose gels with a lambda DNA standard.

### SLAF library construction and high-throughput sequencing

SLAF-seq was used to genotype 100 individuals, and the two parents, as previously described [18], with small modifications. First, a pilot SLAF experiment was performed to establish the conditions to optimize SLAF yield. The enzymes and sizes of restriction fragments were evaluated using training data. Three criteria were considered: i) The number of SLAFs must be suitable for the specific needs of the research project; ii) the SLAFs must be evenly distributed through the sequences to be examined; and iii) repeated SLAFs must be avoided. Next, based on the result of the pilot experiment, the SLAF library was constructed as follows. Genomic DNA was first incubated at 37 °C with Hae III and Hpy166II [New England Biolabs (NEB), Ipswich, MA, USA] for complete digestion, a single-nucleotide A overhang was added to the digested fragments using the Klenow Fragment (3′ → 5′ exonuclease) (NEB) and dATP at 37 °C. Duplex Tag-labeled Sequencing adapters (PAGE purified, Life Technologies) were then ligated to the A-tailed DNA using T4 DNA ligase. The PCR reaction was performed using diluted restriction-ligation samples, dNTP, Q5® High-Fidelity DNA polymerase and PCR primers: AATGATACGGCGACCACCGA and CAAGCAGAAGACGGCATACG (PAGE purified, Life Technologies). The PCR products were purified using Agencourt AMPure XP beads (Beckman Coulter, High Wycombe, UK) and pooled. The pooled sample was separated by electrophoresis through a 2 % agarose gel. Fragments of 264–464 bp (with indexes and adaptors) were excised, purified using QIAquick Gel Extraction Kit (QIAGEN) and diluted for pair-end sequencing on an Illumina Highseq™ 2500 sequencing platform (Illumina, Inc; San Diego, CA, USA) at Biomarker Technologies Corporation in Beijing (http://www.biomarker.com.cn/). Real-time monitoring was performed for each cycle during sequencing. The ratio of high quality reads with quality scores greater than Q30 (representing a quality score of 20, indicating a 1 % chance of an error, and thus 99 % confidence) in the raw reads and the guanine-cytosine (GC) content were calculated for quality control.

### SLAF-seq data grouping and genotype definition

All SLAF pair-end reads with clear index information were clustered based on sequence similarity, as detected by BLAT (−tileSize = 10 –stepSize = 5) [39]. Sequences with over 90 % identity were grouped in one SLAF locus, as described by Sun et al. [18]. Alleles were defined in each SLAF using the minor allele frequency (MAF) evaluation. The mapping population is DH; therefore, one locus contains at most two SLAF tags, so groups containing more than two tags were filtered out as repetitive SLAFs. In this study, SLAFs with a sequence depth of less than 100 were defined as low-depth SLAFs and were filtered out. SLAFs with two tags were identified as polymorphic SLAFs and considered as potential markers. Polymorphic markers were classified into eight segregation patterns (ab × cd, ef × eg, hk × hk, lm × ll, nn × np, aa × bb, ab × cc and cc × ab). Given that the map population is DH, the study only used those SLAF markers whose segregation patterns were aa × bb for genetic map construction.

### Segregation analysis and bin-map construction

Marker segregation ratios were calculated using the chi-square test, and markers showing significant segregation distortion (*P* < 0.05) were excluded from the map construction. The recombination rates between markers were calculated using JoinMap 4.0 software [40] and the genetic map was constructed using a modified logarithm of odds (mLOD) threshold ≥ 7.0 (http://www.kyazma.nl/index.php/mc.JoinMap/sc.FAQ) and a maximum recombination fraction of 0.4. All high quality and non-distorted SLAFs markers were allocated into five LGs based on their locations on chromosomes. Considering that next generation sequencing data may cause many genotyping errors and deletions, which could greatly reduce the quality of high-density linkage maps, the HighMap Strategy was used to order SLAF markers and correct genotyping errors within the LGs [41]. The MSTmap algorithm was used to order the SLAFs markers [42] and the SMOOTH algorithm [43] was used to correct genotyping errors following marker ordering. All linkage groups underwent these procedures: primary marker orders were first obtained by their location on chromosomes, according to the relationship between ordered markers, and genotyping errors or deletion were corrected by SMOOTH algorithm; after that MSTmap was used to order the map and again SMOOTH was taken to correct the new ordered genotypes. After four or more cycles, five high-quality maps were obtained. Map distances were estimated using the Kosambi mapping function [44].

The obtained genetic maps contained many redundant markers that provided no new information, but increased the computational requirements of mapping. To address these issues, the bin-markers approach developed by Huang et al. (2009) was used to combine all the markers in the same locus into one bin [28]. A “bin” means a group of markers with a unique segregation pattern that is separated from adjacent bins by a single recombination event. Using this method, five high-quality bin-maps of *P. haitanensis* were obtained.

### Phenotypic data analysis

The DH population and parents were evaluated in randomized complete block design with three biological replicates, each composed by 10 gametophytic blades per flask. Each biological replicate was evaluated in an identical but independent experiment performed on a seven-day interval. First, the conchocelis of 102 lines (include100 DH lines and their 2 parental lines) were induced to release conchospores, respectively. Second, the conchospores of each line were collected in separate 300-mL flask containing 200-mL culture medium, and cultured with aeration in an incubator at 21 ± 1 °C under 50-60 μmol•photons m^-2^ s^-1^ (12 L: 12D) to develop into gametophytic blades, with the culture medium renewed every 3 days. Third, after the lengths of gametophyte blades were 4.0 ± 0.2 cm, 10 healthy and integrated gametophytic blades derived from each line were randomly selected and place into 1000-mL flasks containing 700-mL culture medium. Culture conditions were the same as described above, but the culture medium was renewed every 2 days. The frond length (FL), width (FW), thickness (FT) and fresh weight (W) of the gametophytic blades were measured after 10 days in culture. The growth rates of frond length and fresh weight were calculated using the formulas:

$$ \mathrm{Frond}\ \mathrm{length}\ \mathrm{growth}\ \mathrm{rate}\ \left(\mathrm{L}\mathrm{G}\mathrm{R}\right) = \left({ \ln \mathrm{L}}_{\mathrm{n}}\kern0.5em \hbox{-} \kern0.5em { \ln \mathrm{L}}_0\right)/\mathrm{n} \times 100\ \% $$

$$ \mathrm{Fresh}\ \mathrm{weight}\ \mathrm{growth}\ \mathrm{rate}\ \left(\mathrm{W}\mathrm{G}\mathrm{R}\right) = \left({ \ln \mathrm{W}}_{\mathrm{n}}\kern0.5em \hbox{-} \kern0.5em { \ln \mathrm{W}}_0\right)/\mathrm{n} \times 100\ \% $$

where L_n_ is the frond length of gametophytic blades that have been cultured for n days (cm), L_0_ is the initial length of the gametophytic blades, W_n_ is the fresh weight of gametophytic blades that have been cultured for n days (mg), and W_0_ is the initial fresh weight of gametophytic blades. All 10 gametophytic blades of each line were measured, and the mean value was calculated by the Microsoft Excel 2010 and was designated as the phenotypic value of each line.

### Quantitative trait locus (QTL) analyses

The mean phenotypic data of three replicates in different trials from all 102 lines (include100 DH lines and their 2 parental lines) were analyzed for frequency distributions, standard errors, coefficient of variation and ANOVA using SPSS 10.0. The winQTLCart program (http://statgen.ncsu.edu/qtlcart/WQTLCart.htm) was used for QTL analysis, and the composite interval mapping (CIM) method [45] was employed to detect any significant associations between each trait and marker loci. Significant LOD thresholds for every trait were calculated by the permutation test of α < 0.05 and *n* = 1000 for significant linkages. Based on these permutations, a LOD score of 2.5 was used as a minimum to declare the presence of a QTL in a particular genomic region.

## Additional files

Additional file 1: Table S1. The genotypes of 4550 LP markers that were mapped onto the genetic map. (XLSX 1645 kb) Additional file 2: Table S2. The genotypes of 6731 LP markers. (XLSX 2652 kb) Additional file 3: Table S3. The markers located on the five linkage groups and their genetic distances. (XLSX 87 kb) Additional file 4: Table S4. The bin markers located on the five linkage groups and their genetic distances. (XLSX 25 kb) Additional file 5:
**Haplotype maps of the five linkage groups.** (RAR 143 kb) Additional file 6:
**Heatmap of the five linkage groups.** (RAR 151 kb) Additional file 7: Table S5. Phenotypic traits of the 100 DH lines and their parents. (XLSX 17 kb)
